# Development of Radiosterilized Porcine Skin Electrosprayed with Silver Nanoparticles Prevents Infections in Deep Burns

**DOI:** 10.3390/ijms232213910

**Published:** 2022-11-11

**Authors:** Mario Alberto Pérez-Díaz, Elizabeth Alvarado-Gómez, María Esther Martínez-Pardo, Miguel José Yacamán, Andrés Flores-Santos, Roberto Sánchez-Sánchez, Fidel Martínez-Gutiérrez, Horacio Bach

**Affiliations:** 1 Laboratorio de Biotecnología, Instituto Nacional de Rehabilitación Luis Guillermo Ibarra Ibarra (INR-LGII), Calzada México Xochimilco No. 289, Colonia Arenal de Guadalupe, Tlalpan, Ciudad de México 14389, Mexico; 2 Laboratorio de Antimicrobianos, Biopelículas y Microbiota, Facultad de Ciencias Químicas, Universidad Autónoma de San Luis Potosí, Av. Dr. Manuel Nava No. 6, Zona Universitaria, San Luis Potosí 78210, Mexico; 3 Banco de Tejidos Radioesterilizados, Instituto Nacional de Investigaciones Nucleares (BTR-ININ), Carretera México-Toluca S/N La Marquesa, Ocoyoacac 52750, Mexico; 4 Applied Physics and Materials Science Department, Core Faculty Center for Materials Interfaces in Research and Applications (MIRA), Northern Arizona University, Flagstaff, AZ 86011, USA; 5 Unidad de Ingeniería de Tejidos Terapia Celular y Medicina Regenerativa, Instituto Nacional de Rehabilitación Luis Guillermo Ibarra Ibarra (INR-LGII), Calzada México Xochimilco No. 289, Colonia Arenal de Guadalupe, Tlalpan, Ciudad de México 14389, Mexico; 6 Escuela de Ingeniería y Ciencias, Departamento de Bioingeniería, Instituto Tecnologico de Monterrey, C. Puente No. 222, Colonia Arboledas Sur, Tlalpan, Ciudad de México 14380, Mexico; 7 Centro de Investigación en Ciencias de la Salud y Biomedicina, Universidad Autónoma de San Luis Potosí, Sierra Leona No. 550, Lomas, San Luis Potosí 28210, Mexico; 8 Division of Infectious Diseases, Department of Medicine, University of British Columbia, 2660 Oak Street, Vancouver, BC V6H 3Z6, Canada

**Keywords:** antibiofilm activity, cytotoxicity, nanoparticle deposition, multidrug-resistant, microwave synthesis, electrospray, burns

## Abstract

Extensive burns represent a significant challenge in biomedicine due to the multiple systemic and localized complications resulting from the major skin barrier loss. The functionalization of xenografts with nanostructured antibacterial agents proposes a fast and accessible application to restore barrier function and prevent localized bacterial contamination. Based on this, the objective of this work was to functionalize a xenograft by electrospray deposition with silver nanoparticles (AgNPs) and to evaluate its antibiofilm and cytotoxic effects on human fibroblasts. Initially, AgNPs were synthesized by a green microwave route with sizes of 2.1, 6.8, and 12.2 nm and concentrations of 0.055, 0.167, and 0.500 M, respectively. The AgNPs showed a size relationship directly proportional to the concentration of AgNO_3_, with a spherical and homogeneous distribution determined by high-resolution transmission electron microscopy. The surface functionalization of radiosterilized porcine skin (RPS) via electrospray deposition with the three AgNP concentrations (0.055, 0.167, and 0.500 M) in the epidermis and the dermis showed a uniform distribution on both surfaces by energy-dispersive X-ray spectroscopy. The antibiofilm assays of clinical multidrug-resistant *Pseudomonas aeruginosa* showed significant effects at the concentrations of 0.167 and 0.500 M, with a log reduction of 1.3 and 2.6, respectively. Additionally, viability experiments with human dermal fibroblasts (HDF) exposed to AgNPs released from functionalized porcine skin showed favorable tolerance, with retention of viability more significant than 90% for concentrations of 0.05 and 0.167 M after 24 h exposure. Antibacterial activity combined with excellent biocompatibility makes this biomaterial a candidate for antibacterial protection by inhibiting bacterial biofilms in deep burns during early stages of development.

## 1. Introduction

The skin is an extensive and complex organ with widely heterogeneous structural and cellular components, such as blood vessels, hair follicles, sebaceous glands, keratinocytes, fibroblasts, and immune cells. All these components should interact during skin repair upon tissue damage [1,2]. In this context, one of the most complex forms of damage caused to the skin occurs when it is burnt. This damage is exacerbated when extensive burns from 30% to 40% of total body surface area (TBSA) occur, which require attention in critical care units, where the first step is to prevent hypovolemic shock resulting from fluid loss [3]. In addition, extensive burns carry an important immunosuppressive factor, which triggers infections by nosocomial and multidrug-resistant (MDR) bacteria.

In burn patients, 75% of deaths worldwide result from infections, with 265,000 deaths yearly. This trend prevails in developing countries, with 90% of cases and a 100% mortality rate when the burn is >40% TBSA [4]. Moreover, burn patients are a considerable burden to the health system. For example, in the UK, around GBP 5.3 billion is spent on wound management yearly [4].

One of the main microorganisms causing nosocomial infections is the opportunistic Gram-negative pathogen *Pseudomonas aeruginosa*. The World Health Organization has identified *P. aeruginosa* as a high-priority pathogen. This pathogen possesses multiple defense mechanisms, such as lectins, protein secretion, pili, exotoxins, and biofilm production, all associated with treatment failure [5,6].

Biofilms provide a structure of protection and resistance to antibiotic treatments, and are difficult for the immune system to act on [7,8]. The infectious processes of MDR bacteria cause therapeutic failures, which extends the stay of patients in hospitals and triggers an increase in morbidity and mortality [9]. To prevent infections and the subsequent development of bacterial biofilms, the use of silver nanoparticles (AgNPs) has been proposed [10,11,12]. The main advantage of using AgNPs lies in the different postulated mechanisms of action, among them are the destabilization and inhibition of cell wall synthesis; generation of reactive oxygen species; an affinity for DNA, RNA, and proteins; and the inhibition of essential enzymes in the metabolic processes of bacteria [13,14].

Using biomaterials combined with metallic NPs should promote a biocompatible environment, allowing the survival of the cells (e.g., fibroblasts) on the surface of the damaged tissue during the healing process [15]. On the other hand, skin substitutes are essential in severe burns (>60% TBSA), whose primary function is to prevent infection and fluid loss. In this context, specialists opt for biological substitutes (69%) over synthetic substitutes due to their high costs [16]. The correct application of skin substitutions or xenografts combined with fluid therapy facilitates the proper stabilization of the patient.

Porcine skin has a structure and properties very similar to human skin, and is compatible as a skin substitution during the healing process of burns [17,18,19]. This study analyzed the functionalization of a biological coverage (porcine xenograft) with AgNPs using electrospray deposition. The technique offers a homogeneous and controlled covering process to generate physical covers that can be applied to extensive and severe skin burns, reducing the risk of bacterial colonization in patients.

## 2. Results and Discussion

In this study, we used AgNP microwave synthesis because of the reduced times in the synthesis compared to other NP synthesis techniques, which are based on the principles of ionic conduction and dipole polarization [20,21]. To evenly distribute the NPs on the surface, we used an electrospray technique because of the size of the drops (micrometers to nanometers), which is controlled stably and simply by adjusting the speed of the fluid and electrical voltage [22]. Furthermore, the solvent is evaporated, remaining only on the surface of the NPs, which avoids excess manipulation and damage to the porcine skin. In addition, it increases biocompatibility since toxic reagents do not remain on their surface [23].

### 2.1. AgNPs Characterization

**Figure 1:**
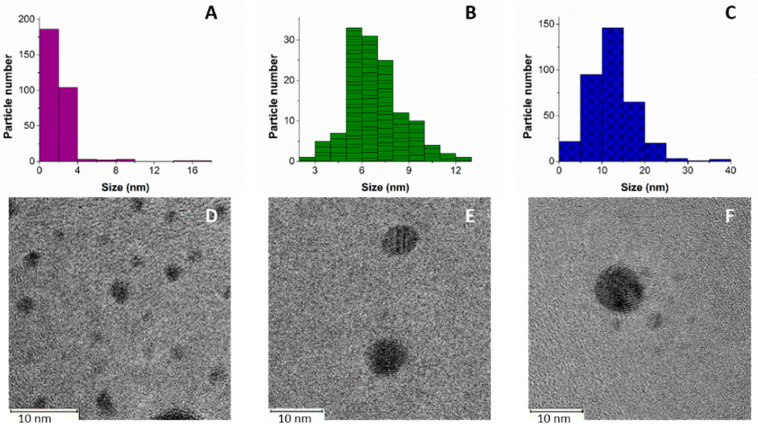
Silver nanoparticles show size-dependent silver nitrate concentration. AgNPs were visualized using size distribution and HRTEM images: (**A**,**D**) 0.055 M AgNO_3_, (**B**,**E**) 0.167 M AgNO_3_, and (**C**,**F**) 0.500 M AgNO_3_.

Microscopic analysis showed that the synthesized AgNPs were spherical, with three different sizes concordant with the concentrations of AgNO_3_ used in their synthesis. The average sizes of the AgNPs were 2.1 ± 1.49, 6.8 ± 1.75, and 12.2 ± 5.3 nm when 0.055, 0.167, and 0.500 M of AgNO_3_ was used, respectively (Figure 1). These results indicate that the size of the AgNPs increased with the concentration of AgNO_3_. The three types of AgNPs were prepared to compare their antibiofilm and cytotoxic effects, because the effect of NP size influences their biological activity [24]. In other studies, similar behaviors of shape and size have been shown for AgNPs synthesized by microwaves, especially with the lowest concentration, demonstrating antifungal and antibacterial activity [25,26].

Nevertheless, the AgNO_3_ concentration affects the nanoparticle size and other experimental conditions, such as the power of induced energy, exposition time, reducing agents, and support surfaces. For example, a recent study was published in which AgNPs were synthesized using the same microwave parameters as performed in our study (1000 W, 10 s). The authors reported smaller sizes (3 nm) compared to our study at 0.500 M AgNO_3_, and the difference is probably the result of a zeolitic surface with a direct effect on the surface [27]. Another study used *Phoenix dactylifera* leaf extract as a reducing agent, and obtained AgNPs with an average size of 40 nm at 300 W for 30 s [28].

### 2.2. Radiosterilized Porcine Skin Covered with Silver Nanoparticles by Electrospray

**Figure 2:**
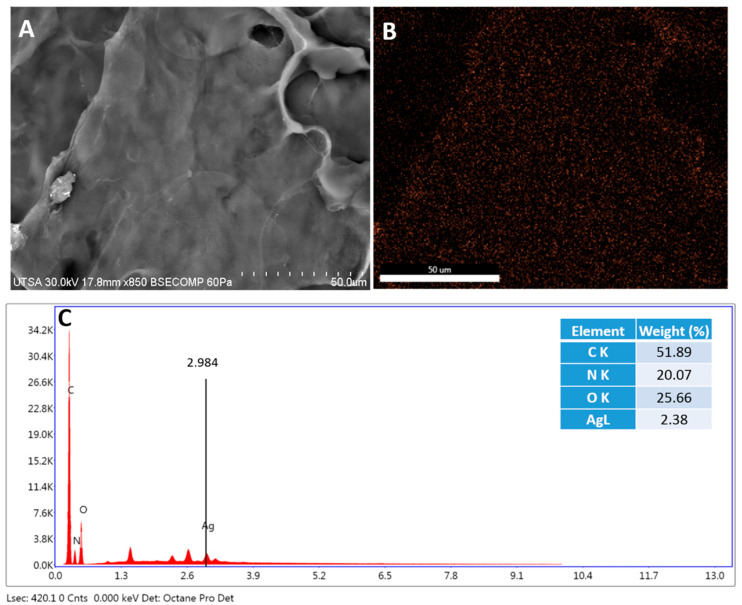
Radiosterilized porcine skin dermis shows a homogeneous distribution of silver nanoparticles. (**A**) SEM image of the dermis; (**B**) EDS mapping of the dermis, orange dots represent the distribution of Ag; and (**C**) analysis of elements expressed as the percentage of elements on the dermis performed by EDS mapping.

**Figure 3:**
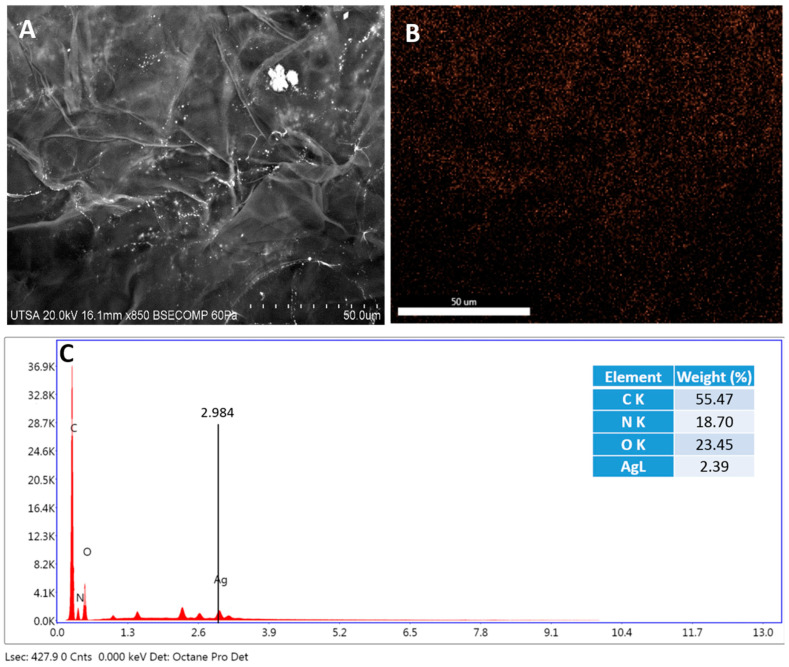
Radiosterilized porcine skin epidermis shows a homogeneous covering of silver nanoparticles. (**A**) SEM image of the epidermis; (**B**) EDS mapping of the epidermis, orange dots represent the distribution of Ag; and (**C**) analysis of elements expressed as the percentage of elements on the epidermis performed by EDS mapping.

The electrospray technique has previously been used to deposit NPs on different surfaces, such as glass or doped silicon [29]. Furthermore, electrospray technology has been used to deposit biomolecules such as drugs, proteins, and DNA on surfaces such as aluminum foil, electrodes, water, and filters, among others [30]. In our study, porcine skin was homogeneously impregnated with AgNPs by electrospray on the dermis (Figure 2) and epidermis (Figure 3).

These results demonstrate that the electrospray technique can cover surfaces more homogeneously than other previously reported techniques, such as immersion and sonication [31]. These reported techniques showed that AgNPs are agglomerated, whereas with the electrospray technique, the size of the droplets is generated on the scale of microns to nanometers, and controlled by voltage and the speed of the fluid [30]. Additionally, the solvent in the electrospray technique is evaporated, eliminating the presence of toxic substances [30].

Another advantage of electrospray is that the integrity of the tissue is maintained, whereas in sonication and immersion treatments, the tissue can be compromised [30].

**Figure 4:**
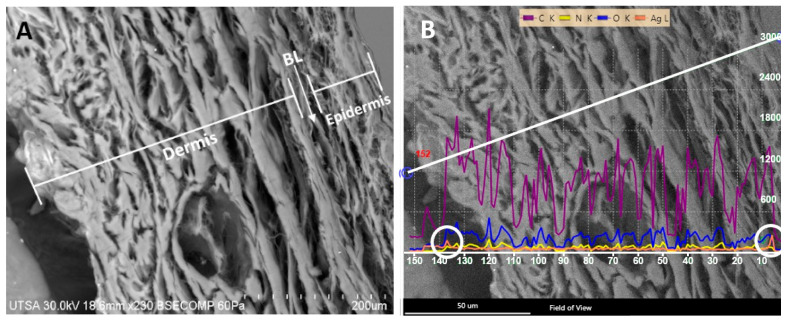
Silver nanoparticles are not retained in the basal lamina. (**A**) SEM image of basal lamina (BL); (**B**) EDS line scan of the basal lamina. The white circles indicate the Ag signal.

We noticed that the AgNPs were not retained in the basal lamina (Figure 4). This finding is aligned with an earlier study that showed that the basal lamina was not stained with Ag during an electron microscopic analysis of the layer [32]. One explanation for this is related to the presence of fibrils extended from the dermis to support the epidermis [33,34]. Thus, the density of the fibrils might allow the passage of the AgNP between the epidermis through the dermis without retention. The fibril density does not follow a homogeneous distribution [35], and the area used in our electron microscopy analysis could have been a less dense area.

Another structure that can contribute to the lack of AgNP retention is bundled microfibrils [32]. Electron microscopy images show that these fibrils are as long as 8–11 nm in diameter [32], potentially allowing the passage of the AgNPs.

**Figure 5:**
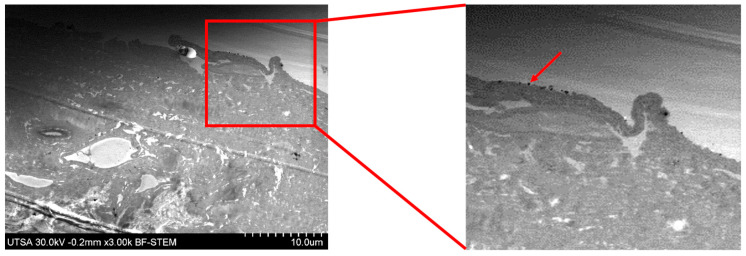
Porcine skin image by STEM. The red arrow shows AgNPs on the skin surface.

To demonstrate the presence of AgNPs on the surface of the epidermis, a section of the porcine skin was analyzed with STEM using the bright field (Figure 5). An amplified visualization of the skin surface showed the presence of black spheres (amplified red square) marked by the red arrow. Another study reported that the covering of pig small intestine submucosa with AgNPs (50 µg/mL) showed promising results in the healing process. In addition, the study evaluated the antibacterial effect of the biological dressing against *Pseudomonas aeruginosa* (ATCC 27853, 1.3 × 10^9^ CFU), where no adverse effects of tissue infection were observed [36].

In addition to tissues impregnated with AgNPs for treating wound infections, there are many reports of alternative materials with the promise of being biocompatible and effective, such as electrospinning dressings. Nevertheless, this technology is not very accessible since fabricating fibers takes a long time, and it is complicated to obtain the ideal conditions for work.

Other studies have reported nanofiber fabrication using electrospinning with a mix of carboxymethyl cellulose, polyvinyl alcohol, and AgNO_3_. The authors of one such study demonstrated the synthesis of AgNPs and nanofibers by SEM, but the size and shape of the AgNPs were not reported [37]. Another study reported the synthesis of a dressing produced from bacterial cellulose with AgNPs synthesized in situ. Results of the synthesis showed AgNPs with spherical shapes ranging between 80 to 100 nm [38].

Lastly, nanofibrous mats from moth tasar fibroin (*Antheraea mylitta*) were produced by electrospinning. The mats were coated in situ with AgNPs using dandelion leaf extract (*Tridax procumbens*). Although a good AgNP distribution was demonstrated, the antibacterial activity against planktonic *P. aeruginosa* was deficient, possibly due to the size of the AgNPs (20–50 nm) [39].

### 2.3. Antibiofilm Activity

**Figure 6:**
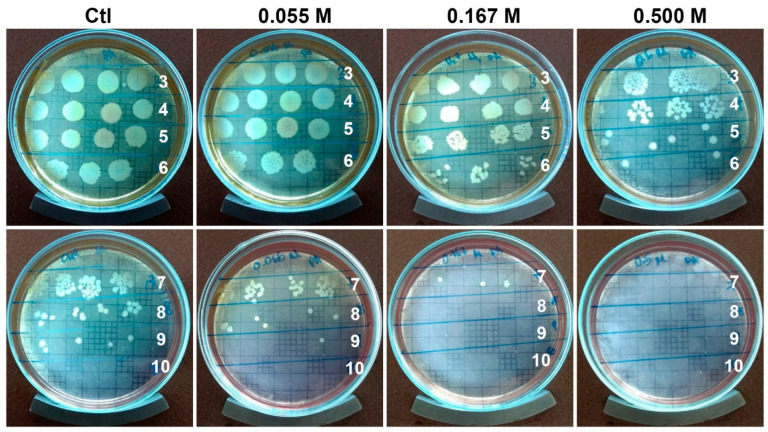
Radiosterilized porcine skin electrosprayed with silver nanoparticles reduces the number of CFU of *Pseudomonas aeruginosa*. Numbers indicate absolute serial dilutions. Ctl, control.

A clinical isolate of an MDR *P. aeruginosa* was used to show the antibiofilm activity of the radiosterilized porcine skin impregnated with AgNP. Our study evaluated the activity of the three AgNPs at the concentrations of 0.055, 0.167, and 0.500 M. After 24 h incubation, the biofilm formed on the impregnated porcine skin was mechanically disrupted. The surviving bacteria were counted (Figure 6).

**Figure 7:**
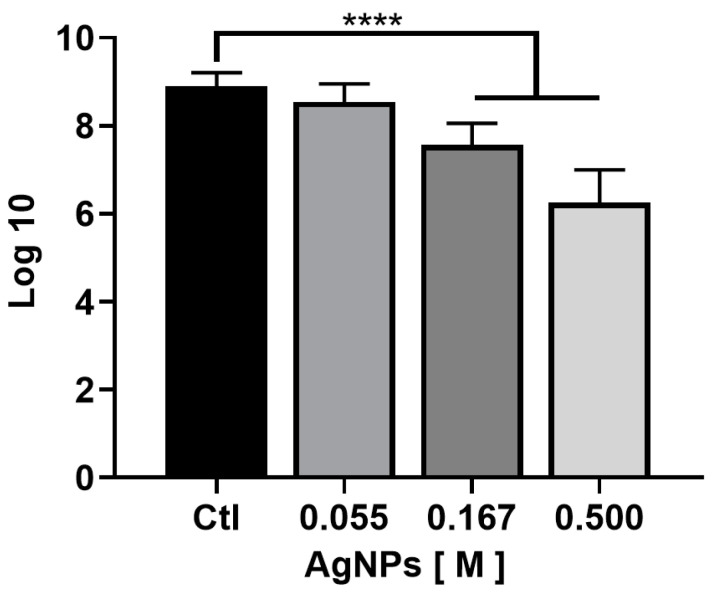
Radiosterilized porcine skin electrosprayed with silver nanoparticles inhibits *Pseudomonas aeruginosa* biofilm formation. Treatment with RPS at 0.167 and 0.500 M of AgNPs significantly inhibits the *Pseudomonas aeruginosa* biofilm formation; **** *p* < 0.0001. Results are expressed as the mean plus or minus SD of three independent experiments.

The results show that the clinical strain was susceptible to the treatments, with a CFU log reduction of 1.3 and 2.6 CFU for the concentrations of 0.167 and 0.500 M (Figure 7). Previous work used a similar radiosterilized pig skin with AgNPs (13 nm) deposited by a sonication technique (40 kHz) [31]. The authors reported antibiofilm activities of approximately 1 CFU log reduction using 125 and 250 µg/mL, and about 2 log reduction using 500 µg/mL against biofilm produced by the pathogen *Staphylococcus aureus* with complete eradication at a concentration of 1000 µg/mL. However, better results were obtained when biofilms of *Stenotrophomonas maltophilia* were evaluated. In this case, the pathogen was more susceptible to complete eradication at 250 µg/mL concentration.

Although our antibiofilm activity was higher at the same AgNP concentration (500 µg/mL), we used an MDR, *P. aeruginosa*, isolated from burn patients.

Recent studies have shown that AgNPs in suspension (20–40 nm) significantly prevent biofilm formation from concentrations of 6.25 µg/mL and the eradication of mature biofilm with a CFU log reduction of 2 at concentrations of 50 µg/mL in *P. aeruginosa* PAO1 [40]. This antibiofilm activity appears superior to our results; however, AgNPs were not deposited on a scaffold surface, as performed in our study. Furthermore, the size of the AgNPs was calculated according to the core, and did not include the addition of compounds after exposing them to an extract of *Cannabis sativa*. In this case, the size of the NPs increased to >100 nm. Thus, the antibiofilm activity may be higher because the biofilm formation occurred without agitation, and the AgNPs and the bacteria settled together. As a result of this sedimentation process, bacteria would have direct contact with the AgNPs.

### 2.4. Viability of Dermal Fibroblast

**Figure 8:**
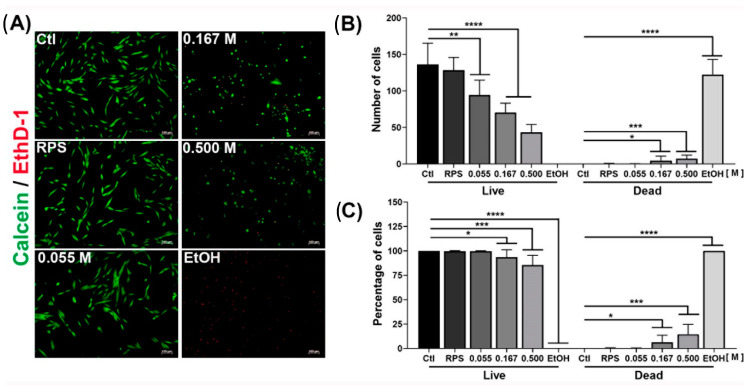
Viability of fibroblasts exposed to electrosprayed silver nanoparticles. The effect of AgNPs from porcine skin on fibroblast viability was measured. (**A**) Micrographs of viable cells (calcein/green) and dead cells (ethidium homodimer/red); (**B**) number of living and dead cells; and (**C**) percentage of living and dead cells. Experiments were performed in triplicate. Untreated cells and ethanol were used as negative and positive controls, respectively. Results are expressed as the mean ± SD. * *p* < 0.05; ** *p* < 0.01; *** *p* < 0.001; **** *p* < 0.0001.

To determine the effect of Ag released from electrosprayed porcine skin on viability in eukaryotic cells, human dermal fibroblast (HDF) from young adult patients undergoing cosmetic surgery was used. After 24 h of exposure to the medium in contact with porcine skin, the corresponding cells presented a behavior similar to the control (Figure 8A), with a viability of 99% for RPS (Figure 8C). For RPS-AgNPs containing the concentrations 0.055, 0.167, and 0.500 M, the treatments showed a viability of 99, 93, and 85%, respectively (Figure 8C). Additionally, the number of live cells (calcein/green) decreased significantly in the three treatments with electrosprayed nanoparticles compared to the control (Figure 8B). The concentration of 0.055 M presented a significant decrease of 25% in the number of living cells. In comparison, the concentrations of 0.167 and 0.500 M decreased the viability considerably to 34 and 55%, respectively (Figure 8B). The results are represented as the number and percentage of cells, since the percentage in both conditions is high. Still, the number of cells decreased when the concentration of AgNPs increased. Our results show that the lowest concentration of AgNPs affects cell viability, since the number of cells decreased, but most of the remaining cells in the scaffold were viable. Even when the number of cells started to decrease from 0.055 M, the morphological changes began to appear at 0.167 M, where the cells showed a rounded morphology, which indicates detachment from the cell culture plate and cell death. For future assays, to evaluate the cytotoxicity of the AgNPs released from the nanomaterial, they could be placed in transwell cell culture plates, and the fibroblasts could be cultured at the bottom; in this case, the cytotoxic effect of the AgNPs released could be evaluated.

Fibroblasts play a fundamental role in regeneration processes since, in the proliferative phase, they promote the formation of granular tissue, which results in the formation of a new extracellular matrix (ECM), ending in the closure of the wound [41]. Thus, wound coverings impregnated with Ag should be considered of limited use since AgNPs can be attacked by reactive oxygen species, which affect cell viability and proliferation [42]. From a therapeutic point of view, dressings with any silver-based component should be limited as an infection-preventive agent in the first 48 h from the onset of skin burn. The Ag composite is removed, and an appropriate treatment continues [43]. Although we observed some cytotoxic effects in fibroblasts, the topical cream used in burn patients showed higher cytotoxicity in dressing when compared to AgNPs [44]. Moreover, some models have been used to evaluate different concentrations of AgNPs, and the results lead to the conclusion that when applying AgNPs at low concentrations in albino mice, no silver was detected in internal organs, nor adverse effects on the area affected by the burn [45], which is consistent with the results obtained in our experiments. Another study showed that in rats with continuous administration of AgNPs for 4 weeks with 100 and 500 mg/kg/day, accumulation of AgNPs in the blood, kidney, liver, and spleen was not detected [45]. One advantage of our study is that AgNPs were impregnated on the PPR, and even when the impregnation could be performed at low concentrations, the release of the AgNPs is expected to be lower, according to our previous results [31].

## 3. Methods

### 3.1. Synthesis and Characterization of AgNPs

AgNPs were synthesized by microwave (MW) using an Ethos EZ Digestion System Microwave (Milestone, 2.5 GHz, sensor ATC400) [46]. Three samples were prepared in glass flasks by weighing the necessary amount of AgNO_3_ (99.99% purity, Sigma-Aldrich, St. Louis, MO, USA). The powder was dissolved in 15 mL of distilled water, affording concentrations of 0.055, 0.167, and 0.500 M. After that, each flask solution was introduced in the MW and irradiated for 10 s at 1000 W. AgNPs were characterized by a High-Resolution Transmission Electron Microscopy (HRTEM) analyses 2010-F JEOL (JEOL Inc., Tokyo, Japan) with a field emission gun operating at 200 kV. Ten images were analyzed, and the average and standard deviation were calculated for each concentration [47].

### 3.2. Covering Radiosterilized Porcine Skin with AgNPs Using Electrospray

Radiosterilized porcine skin was obtained and processed by the Radiosterilized Tissue Bank at National Institute of Nuclear Research, as described previously [48]. Then, the RPS was cut into 2 × 2 cm^2^ in a sterile laminar flow hood using all the accessory tools (scissors, aluminum foil, tweezers, etc.) and sterilized by UV light (λ = 325 nm) for 15 min. A cleaned chamber was lined with aluminum foil, and a universal support was placed inside the chamber. The Ultrasonic Atomizer Generator CSA40 was also placed inside the chamber and was adjusted with pressure tweezers on the universal support. A sterile Petri dish (without a lid) with a square of sterile aluminum foil was placed on the base of the universal support, and a square of porcine skin. The distance between the atomizer and the porcine skin was kept at 2 cm. A hose was connected to the atomizer, and a syringe loaded with the AgNP suspensions with a flow rate of 110 µL/min was used. The three concentrations of AgNPs were deposited on the dermis and epidermis of several samples of radiosterilized porcine skin by the electrospray technique that consisted of applying 0.04 A through an Ultrasonic Homogenizer Power Supply MSK-SP-01A (MTI Co., New York, NY, USA). Only one pulsation to the power supply was necessary to deposit the AgNPs on each side of the sample. After covering one piece of skin, the square of sterile aluminum foil was changed. Between each concentration, the syringe and the hose were purged with isopropanol.

### 3.3. Characterization of Radiosterilized Porcine Skin Covered with AgNPs

The microscopy techniques were performed using radiosterilized porcine skin covered with AgNPs (RPS-AgNPs) at 0.055 M. A Hitachi SU-1510 Scanning Electron Microscope (SEM) was used in VP mode to obtain the images. Analyses were performed by Energy-Dispersive X-ray Spectroscopy (EDS) with a solid-state EDAX EDS detector to determine chemical elements, especially to detect Ag between the dermis and epidermis (basal lamina) of porcine skin samples. For the analysis by Transmission Electron Microscopy (TEM), a small piece of RPS-AgNPs was rinsed with PBS buffer and centrifuged for 5 min at 3000 rpm. Then, 1 mL of 1% osmium tetroxide (OsO_4_) diluted with buffer was added to the skin sample to stain for a 24 h period. Afterward, the sample was centrifuged for 5 min at 3000 rpm, the supernatant was discarded, and the sample was dehydrated in ethanol at different concentrations (25, 50, 75, 95, and 100%). Additionally, the sample was dehydrated with propylene oxide. Finally, the RPS was mounted on a resin (LX112; Ladd Research Industry, Williston, VT, USA) and solidified for 48 h at 60 °C. Porcine skin cuts were produced with an ultramicrotome (Leica Ultracut, UCT, Hayward, CA, USA) and a 45° diamond blade [49]. Ultrathin sections (approximately 95 nm) were placed on a C grid and visualized in a Hitachi S 5500 scanning electron microscope.

### 3.4. Bacterial Strain Used in This Study

The *P. aeruginosa* clinical isolate was obtained from the skin wound of a hospitalized patient at the Central Hospital, Dr. Ignacio Morones Prieto Hospital, San Luis Potosi, Mexico using an approved protocol (102-16) by the Ethics Committee of the hospital. The identification and resistance profile of the isolated strain was performed with a VITEK^®^ automated system. The antibiogram of the clinical isolate was as follows (in µg/mL): amikacin ≥ 64 (R), ampicillin/sulbactam 16 (R), cefepime ≥ 64 (R), ceftazidime ≥ 64 (R), ciprofloxacin ≥ 2 (R), doripenem ≤ 2 (S), gentamicin ≥ 16 (R), imipenem ≤ 2 (S), meropenem ≤ 2 (S), piperacillin/tazobactam ≤ 16/4 (S), and trimethoprim/sulfamethoxazole ≤ 20 (R).

A stock solution of the pathogen was stored at −20 °C for future work. When necessary, a 200 µL aliquot was added to 800 µL of Mueller–Hinton broth (B&D) and incubated for 24 h with continuous shaking.

### 3.5. Evaluation of the Antibiofilm Activity

A stored vial of *P. aeruginosa* was incubated for 4 h. Then, the microbial suspension was used to inoculate Petri dishes with soy trypticase agar (STA, B&D) and allowed to grow for 24 h. The concentration of the used microorganism was 1.5 × 10^8^ CFU/mL (0.5 McFarland scale).

The colony biofilm technique was used to analyze the inhibition of biofilm development. A piece of RPS-AgNPs was cut into 1 × 1 cm and placed on STA under sterile conditions. An aliquot of 10 μL of *P. aeruginosa* inoculum was placed on the surface of RPS-AgNPs. RPS without AgNPs was used as a negative control. Finally, the samples were incubated at 37 °C for 24 h) and the antibiofilm activity was evaluated in triplicate. After the incubation period, the RPS-AgNPs and RPS were removed from STA and transferred to a test tube with 9 mL of sterile saline solution to perform serial dilutions. Once all the dilutions were achieved, 10 μL of each dilution was plated on STA for colony-forming unit counting at 37 °C for 24 h [50].

### 3.6. Isolation of Dermal Fibroblast

Human dermal fibroblasts were isolated from patients undergoing cosmetic surgery. Patients signed a consent document, and the study was performed by the principles embodied in the Declaration of Helsinki. The Ethics Committee approved the protocol of the Instituto Nacional de Rehabilitación Luis Guillermo Ibarra (INR 20/19 AC). Fibroblasts were isolated from a biopsy, and were measured, weighed, and washed with sterile PBS containing 10% antibiotic/antimycotic (×3) for 5 min. The excess fat was removed, and parallel cuts were produced throughout the sample. Then, the protease dispase was added (3 mg/mL) and incubated at 37 °C for 40 min. The dermis was mechanically detached and treated with type I collagenase (10 mg/mL) in DMEM-F12 (Invitrogen, Waltham, MA, USA). Subsequently, the biopsy was incubated at 37 °C for 2 h under shaking (210 rpm). Aliquots of the supernatant were taken every 30 min, and the enzyme was inactivated DMEM-F12 supplemented with fetal bovine serum. The cells were passed through a Falcon^®^ filter of 70 μm and centrifuged (1500 rpm for 5 min). The obtained cells were resuspended in supplemented DMEM-F12 and seeded in a conventional T-25 culture flask at a density of 10,000 cells/cm^2^.

### 3.7. Viability Assay of Dermal Fibroblast

For viability assays, HDFs were exposed to the released Ag from RPS-AgNPs scaffolds. Fibroblasts were used after the second passage (10,000 cells/cm^2^) and seeded in triplicate in 12-well culture plates. Each well was supplemented with 2 mL of DMEM-F12 with 10% fetal bovine serum (FBS) and 1% antibiotic/antimycotic. Then, the cells were incubated at 37 °C and supplemented with 5% CO_2_ for 24 h. In parallel, a piece of 1 cm^2^ of RPS and RPS-AgNPs was placed in triplicate in 12-well culture plates, which were supplemented with 1 mL of DMEM-F12 with 10% FBS and 1% antibiotic/antimycotic. After 24 h of release, the pieces were removed from the culture plates, and the medium was stored under sterile conditions for later use. RPS pieces were used as a negative control.

The supernatants of the growing HDF were discarded, and the cells were washed with PBS (×3). Then, the collected supernatant from the RPS-AgNPs was added to the fibroblasts for another 24 h to evaluate their cytotoxic effects.

To assess the effect of RPS-AgNPs scaffolds on the viability of HDFs, a LIVE/DEAD^®^ Viability/Cytotoxicity Kit (Molecular Probes) was used according to the manufacturer’s instructions. The viability of the cells was estimated at 24 h of culture. Samples were analyzed on an Axio Observer A1 fluorescence microscope (Carl Zeiss, Jena, Germany). Finally, the cell count of each treatment was carried out using ImageJ software.

### 3.8. Statistical Analysis

Experiments were performed in triplicate, and a one-way ANOVA was applied using GraphPad Prisma Ver. 8.0.1 program. A *p*-value < 0.05 was considered significant, and the results are reported as the mean ± SD.

## 4. Conclusions

In this study, the first approach to electrospray coating with silver nanoparticles of a temporary biological cover was achieved to treat deep burns. The impregnation started from a green microwave synthesis, where three different sizes of nanoparticles, depending on the concentration of the precursor agent (HNO_3_), were obtained. The homogeneous electrospray functionalization of biomaterials guarantees over other experimental models, such as ultrasound, a technique that can be standardized to obtain reproducible results in the clinical/biological area of medical devices. In this context, the antibiofilm experiments show a homogeneous trend dependent on the concentration of AgNPs in the reduction of the bacterial load, where significant effects were observed from 0.167 M. Although a total reduction was not obtained, silver-based antimicrobial covers are focused on preventing the development of biofilms with lower bacterial loads (<10^5^ CFU), since in the hospital setting burns are debrided and washed before the use of covers. In parallel, in the viability experiments with human dermal fibroblasts (HDF), the same trend in effects was observed as in the antibiofilm assays, where the porcine skin coating significantly reduced the number of live cells (34%) at the concentration of 0.167 M. Additionally, a change in cell morphology was observed, which can affect cell metabolism, limiting essential functions such as adhesion, proliferation, and migration. This narrow behavior between the antimicrobial activity and the effect of reducing viability poses silver-based coatings in burn therapy as transitory agents, applied only for a limited time in the stages where the wound is susceptible to bacterial contamination.

The final application of this biological material is intended to be developed in multiple stages, firmly adhering to hygiene and quality standards in clinical practice. The impregnation of the biological material occurs after the radiosterilization process and before its application in patients. The biological material will be treated as a medical device, coated by electrospray in an area free of contaminating particles and pathogenic agents, with a quality equal to or greater than that of a surgical room. Impregnated materials can be produced and stored for later use. However, according to the relevant Regulatory Offices, the product must have an expiration date.

## Data Availability

Not applicable.
